# The Overexpression of FEN1 and RAD54B May Act as Independent Prognostic Factors of Lung Adenocarcinoma

**DOI:** 10.1371/journal.pone.0139435

**Published:** 2015-10-02

**Authors:** Jau-Chung Hwang, Wen-Wei Sung, Hung-Pin Tu, Kun-Chou Hsieh, Chung-Min Yeh, Chih-Jung Chen, Hui-Chun Tai, Chao-Tien Hsu, Grace S. Shieh, Jan-Gowth Chang, Kun-Tu Yeh, Ta-Chih Liu

**Affiliations:** 1 Graduate Institute of Clinical Medicine, College of Medicine, Kaohsiung Medical University, Kaohsiung, Taiwan; 2 Department of Pathology, Lin Shin Hospital, Taichung, Taiwan; 3 School of Medicine, Chung Shan Medical University, Taichung, Taiwan; 4 Department of Medical Education, Chung Shan Medical University Hospital, Taichung, Taiwan; 5 Department of Medical Technology, Jen-Teh Junior College of Medicine, Nursing and Management, Miaoli, Taiwan; 6 Department of Public Health and Environmental Medicine, School of Medicine, College of Medicine, Kaohsiung Medical University, Kaohsiung, Taiwan; 7 Division of Thoracic Surgery, Department of Surgery, E-Da Hospital, Kaohsiung, Taiwan; 8 Department of Surgical Pathology, Changhua Christian Hospital, Changhua, Taiwan; 9 Department of Pathology, E-Da Hospital and I-SHOU University, Kaohsiung, Taiwan; 10 Institute of Statistical Science, Academia Sinica, Taipei, Taiwan; 11 Department of Laboratory Medicine, and Center of RNA Biology and Clinical Application, China Medical University Hospital, China Medical University, Taichung, Taiwan; 12 Division of Hematology/Oncology, Department of Internal Medicine, Kaohsiung Medical University Hospital, Kaohsiung, Taiwan; University of Nebraska Medical Center, UNITED STATES

## Abstract

Synthetic lethality arises when a combination of mutations in two or more genes leads to cell death. However, the prognostic role of concordant overexpression of synthetic lethality genes in protein level rather than a combination of mutations is not clear. In this study, we explore the prognostic role of combined overexpression of paired genes in lung adenocarcinoma. We used immunohistochemical staining to investigate 24 paired genes in 93 lung adenocarcinoma patients and Kaplan-Meier analysis and Cox proportional hazards models to evaluate their prognostic roles. Among 24 paired genes, only FEN1 (Flap endonuclease 1) and RAD54B (RAD54 homolog B) were overexpressed in lung adenocarcinoma patients with poor prognosis. Patients with expression of both FEN1 and RAD54B were prone to have advanced nodal involvement and significantly poor prognosis (HR = 2.35, P = 0.0230). These results suggest that intensive follow up and targeted therapy might improve clinical outcome for patients who show expression of both FEN1 and RAD54B.

## Introduction

Lung cancer is one of the most common cancers in the world and is a leading cause of cancer death in men and women in Taiwan [1]. A low detection rate of early stage lung cancer results in poor prognosis, with an overall 5-year survival of approximately 15% [1–3]. Pathologic aspects indicate that the two major types of lung cancer—adenocarcinoma and squamous cell carcinoma—have different clinical behaviors, therapeutic strategies, and even prognostic markers [4–7]. These two cancer types also have dissimilar risk factors and differ in the activation of their oncogenic pathways [7, 8]. For these reasons, the discovery of novel markers to predict prognosis and develop personal therapy for cancer patients could contribute to better clinical outcomes.

Cancers form through multiple steps and alteration of multiple signaling pathways; one hallmark is the accumulation of numerous genetic abnormalities in multiple genes [7, 9]. Therefore, the use of numerous prognostic markers as personal therapy was found to improve the outcome of lung cancer patients who fall into different clinicopathological subgroups. One successful model is the use of tyrosine kinase inhibitors in treating lung cancer patients with EGFR mutations [7]. In addition to EGFR-associated signaling pathways, complementary molecular therapeutic approaches that involve simultaneously targeting distinct pathways have potential benefit. Among these markers, synthetic lethality (SL) genes were proposed as novel targets for cancer therapy [10].

SL arises when a combination of mutations in two or more genes leads to cell death, while a mutation in only one of these genes does not (the single mutation by itself is therefore said to be viable) [10, 11]. The potential impact of this recent recognition of SL has prompted exploration of cell signaling from the aspect of SL in different cancer types [12–16]. In lung cancer, the use of siRNA-based SL screens and fragment-based small molecule screens has implicated a therapeutic role for Ras-pathway targeted treatments [17–19]. In particular, a combination of ATR suppression and oncogenic Ras causes a synergistic and dose-dependent increase in genomic instability resulting in SL [19, 20]. In addition to Ras, other genes such as BRAF, KEAP1, PARP, JNK, STAT3, BRG1, and DNA-repair genes also represent novel targets for exploiting SL in the development of lung cancer therapies [21–27]. These results suggested a potential role for SL genes in cancer therapy.

The possibility that SL might contribute to new therapeutic strategies could lead to improved clinical outcome. However, the prognostic role of concordant overexpression of SL genes in protein level rather than a combination of mutations is not clear and still requires investigation. The concept of using immunohistochemistry (IHC) staining to investigate the prognostic role of synthetic lethal genes was proposed previously [11]. In the present study, we analyzed 24 paired genes by IHC staining in 93 lung adenocarcinoma patients to explore the role of concordant overexpression of paired SL genes as prognostic biomarkers in this cancer.

## Materials and Methods

### Ethics statement

The study was approved by the Institutional Review Board and the Ethics Committee of the Changhua Christian Hospital, Changhua, Taiwan (IRB no. 121228). The data were analyzed anonymously, and informed consent from the participants was waived by the Institutional Review Board and the Ethics Committee of the Changhua Christian Hospital.

### Study subjects

A total of 93 patients with lung adenocarcinoma were examined in this study. Surgically resected tumor tissues from patients with confirmed histological diagnosis were collected at Changhua Christian Hospital between 1998 and 2010. Cancers were staged according to the AJCC Cancer Staging Manual (7^th^ edition). Clinical data including gender, age, stage, T, N, and M stages, and follow-up information were obtained from medical records and the cancer registry.

### Immunohistochemistry staining and evaluation of STEAP1 immunoreactivity

IHC staining was performed at department of pathology, Changhua Christian Hospital. Tumor tissue was taken from paraffin blocks and used to construct tissue microarrays composed of tumor tissue and peri-tumoral lung tissue. Antibodies for 24 biomarkers using 22 different biomarkers selected from a literature search were used for the IHC study of tumor tissue (S1 Table). A mouse monoclonal anti-FEN1 (Flap endonuclease 1) antibody (1: 400 dilution, ab462, Abcam Ltd.) and a mouse monoclonal RAD54B (1:60 dilution, sc-101234, Santa Cruz) were used for IHC staining according to the manufacturer’s instructions, the specificity of these antibodies was also confirmed [11]. Each tissue microarray core on the slides was interpreted by 2 pathologists. Staining localized to the cell membrane, cytoplasm, and nucleus was graded on a 0 to 3 intensity scale (0, negative; 1, weakly positive; 2, moderately positive; 3, strongly positive). Positivity was defined as more than 5% of the tumor cells stained by the antibody.

### Statistical analysis

Survival curves were estimated by the Kaplan-Meier product-limit method, and survival distributions were compared across FEN1 and RAD54B expression groups using the log-rank test. We used time-dependent Cox proportional hazards modeling by univariate and multivariate analysis to estimate the hazard ratio (HR) of incident lung cancer associated with the FEN1 and RAD54B expression during the cohort follow-up. Results were presented as hazard ratio (HR) and 95% confidence intervals (95% CI). Differences between categories of FEN1 and RAD54B expression and lung cancer death patients with respect to continuous variables like age were tested by one-way analysis of variance (ANOVA) or linear correlation. Categorical variables were analyzed by a chi-square test. The Cochran-Armitage test for trend was applied for linear correlation between categories of FEN1 and RAD54B expression and categorical variables. Statistical analysis was performed employing the commercial software packages SAS 9.3 (SAS Institute Inc., Cary, North Carolina, USA) and Statistical Package for the Social Sciences, Version 19.0 (SPSS Inc., Chicago, IL, USA).

## Results

### Clinicopathological characteristics and expression of FEN1 and RAD54B in the study subjects

In total, 93 patients with lung adenocarcinoma were included in this study. The clinicopathological characteristics of the study population are shown in Table 1. The mean age was 64.3 ± 12.5 years (mean±SD) and the gender ratio was 0.86: 1.00 (female: male). Thirty-one (33.3%) patients were never smokers, 15 (16.1%) patients were ever smokers, and 47 patients had missed smoking data. The distribution of patients according to TNM stage is listed in Table 1. Most of our cases were in early T stage (81.7% in T1+T2) and early N stage (61.3% in N0). Of the 93 patients, 36 had stage I, 11 had stage II, 23 had stage III, and 23 had stage IV tumors. Twenty-three patients had metastasis at diagnosis. Forty-five patients underwent CT and 67 patients underwent RT after surgery.

**Table 1:** Clinicopathological characteristics of 93 lung adenocarcinoma patients.

	Patients, n (%)
Total number	93
Age (mean ± SD), years	64.3 ± 12.5
Gender male	
Female	43 (46.2)
Male	50 (53.8)
Smoking	
Ever smoke	15
Never smoke	31+miss data (47)
T value	
T1	37 (39.8)
T2	39 (41.9)
T3	12 (12.9)
T4	5 (5.4)
N value	
N0	57 (61.3)
N1	6 (6.5)
N2	24 (25.8)
N3	6 (6.5)
M value	
M0	70 (75.3)
M1	23 (24.7)
Stage	
1	36 (38.7)
2	11 (11.8)
3	23 (24.7)
4	23 (24.7)
CT	
Yes	45 (48.4)
No	48 (51.6)
RT	
Yes	67 (72.0)
No	26 (28.0)

**Fig 1:**
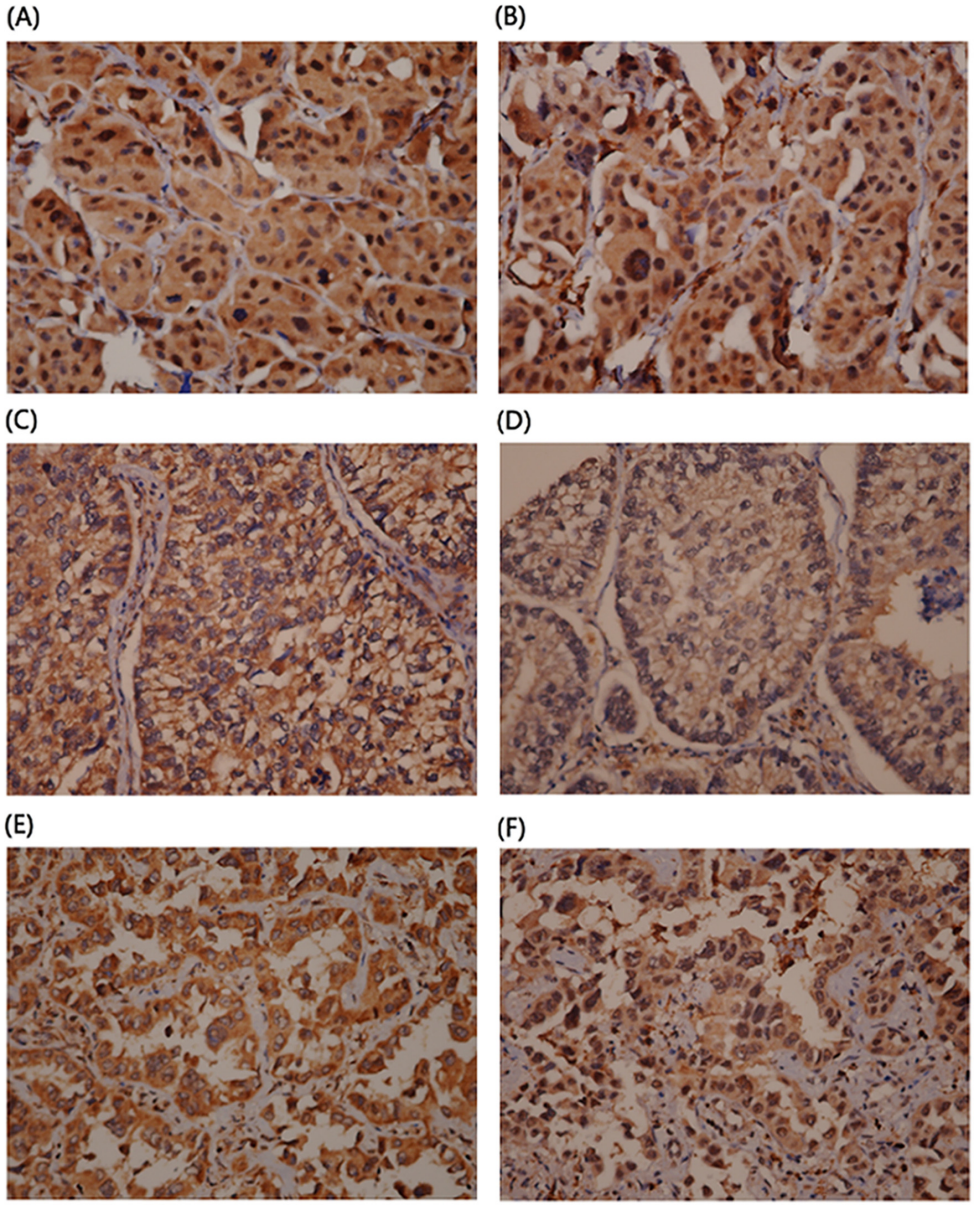
Representative IHC staining of FEN1 and RAD54B in lung adenocarcinoma tissues. (a) and (b): Both positive FEN-1 and RAD54B nuclear staining; (c)and (d): Both negative FEN-1 and RAD54B nuclear staining; (e)and (f): Negative FEN-1 and positive RAD54B nuclear staining.

FEN1 and RAD54B expression was evaluated by IHC staining. Fig 1 shows representative staining results for FEN1 and RAD54B in tumor specimens. Further evaluation of the relationship of FEN1 and RAD54B expression and clinicopathological characteristics revealed no significant association between expression and characteristics including gender, stage, TNM stage, CT, and RT (Table 2). Interestingly, a significant trend was seen between advanced N stage of patients with high FEN1 and RAD54B expression when compared with patients with low or intermediate expression (p for trend = 0.0232, Table 3). These data suggested that FEN1 and RAD54B expression might contribute to nodal metastasis and result in poor prognosis.

**Table 2:** Relationships of FEN1 and RAD54B expression with clinical parameters in lung adenocarcinoma patients.

		FEN1			RAD54B		
Parameters	Case number	0	1–2	p value	0	1–2	p value
Age(SD), year	93	64.4 (11.6)	64.3 (13.5)	0.9701	65.5 (11.0)	62.5 (14.6)	0.2735
Gender, n(%)							
Female	50 (53.8)	24 (50.0)	26 (42.2)		28 (48.3)	15 (42.9)	
Male	43 (46.20	24 (50.0)	19 (57.8)	0.4522	30 (51.7)	20 (57.1)	0.6116
Stage, n(%)							
I	36 (38.7)	20 (41.7)	16 (35.6)		24 (41.4)	12 (34.3)	
II	11 (11.8)	3 (6.3)	8 (17.8)		8 (13.8)	3 (8.6)	
III	23 (24.7)	12 (25.0)	11 (24.4)		14 (24.1)	9 (25.7)	
IV	23 (24.7)	13 (27.1)	10 (22.2)	0.3827	12 (20.7)	11 (31.4)	0.6091
T value, n(%)							
1	37 (39.8)	22 (45.8)	15 (33.3)		28 (48.3)	9 (25.7)	
2	39 (41.9)	16 (33.3)	23 (51.1)		20 (34.5)	19 (54.3)	
3	12 (12.9)	7 (14.6)	5 (11.1)		7 (12.1)	5 (14.3)	
4	5 (5.4)	3 (6.3)	2 (4.4)	0.3885	3 (5.2)	2 (5.7)	0.1771
N value, n(%)							
0	57 (61.3)	32 (66.7)	25 (55.6)		38 (65.5)	19 (54.3)	
1	6 (6.5)	2 (4.2)	4 (8.9)		4 (6.9)	2 (5.7)	
2	24 (25.8)	12 (25.0)	12 (26.7)		13 (22.4)	11 (31.4)	
3	6 (6.5)	2 (4.2)	4 (8.9)	0.5522	3 (5.2)	3 (8.6)	0.6651
M value, n(%)							
0	70 (75.3)	36 (75.0)	34 (75.6)		46 (79.3)	24 (68.6)	
1	23 (24.7)	12 (25.0)	11 (24.4)	0.9505	12 (20.7)	11 (31.4)	0.2449
CT, n(%)							
No	45 (48.4)	23 (47.9)	22 (48.9)		30 (51.7)	15 (42.9)	
Yes	48 (51.6)	25 (52.1)	23 (51.1)	0.9253	28 (48.3)	20 (57.1)	0.4071
RT, n(%)							
No	67 (72.0)	33 (68.8)	34 (75.6)		42 (72.4)	25 (71.4)	
Yes	26 (28.0)	15 (31.3)	11 (24.4)	0.4649	16 (27.6)	10 (28.6)	0.9183

**Table 3:** Clinical effect of FEN1 and RAD54B in lung cancer death patients.

	FEN1 –/RAD54B -	Other	FEN1 +/RAD54B +	p	p-trend
Total number	15	13	18		
Age (SD), years	68.2 (10.5)	65.7 (12.0)	60.2 (17.4)	0.2517	0.1017
Male, n(%)	11 (73.3)	4(30.8)	10 (55.6)	0.0780	0.3565
Tumor grade, n(%)					
Well	3 (20.0)	3 (23.1)	2 (11.1)		
Moderate	9 (60.0)	9(69.2)	13 (72.2)		
Poor differentiated	3 (20.0)	1 (7.7)	3 (16.7)	0.8229	0.7472
T, n(%)					
1+2	11 (73.3)	10 (76.9)	15 (83.3)		
3+4	4 (26.7)	3 (23.1)	3 (16.7)	0.8224	0.4843
N, n(%)					
0	11 (73.3)	6 (46.1)	6 (33.3)		
1+2+3	4 (26.7)	7 (53.9)	12 (66.7)	0.0691	0.0232
M, n(%)					
0	7 (46.7)	10 (76.2)	11 (61.1)		
1	8 (53.3)	3 (23.8)	7 (38.9)	0.2622	0.4367
Stage, n(%)					
1+2	5 (33.3)	5 (38.5)	5 (27.8)		
3+4	10 (66.7)	8 (61.5)	13 (72.2)	0.8198	0.7156
RT CT, n(%)					
RT-/CT-	4 (26.7)	3 (23.8)	6 (33.3)		
Other	11 (83.3)	10 (76.2)	12 (66.7)	0.8108	0.6550

### Overexpression of FEN1 and RAD54B is associated with poor survival in lung adenocarcinoma patients

We further examined the role of FEN1 and RAD54B in the prognosis of lung adenocarcinoma patients by analyzing the clinical outcome with respect to gene expression levels. Overall survival data were collected for all 93 patients. The mean and median follow-up times after surgery were 4.31 years. During the survey, 46 (49.5%) patients died. We confirmed the reliability of the clinical data by using a Cox regression model to evaluate the prognostic role of disease stage. As expected, patients with advanced stage had poorer clinical outcomes when compared with patients diagnosed with stage I disease (Multivariate analysis: HR = 1.55, 95% CI = 0.49–4.93, P = 0.4596 for stage II; HR = 2.32, 95% CI = 1.01–5.35, P = 0.0473 for stage III; HR = 3.06, 95% CI = 1.426.59, P = 0.0.004 for stage IV).

**Fig 2:**
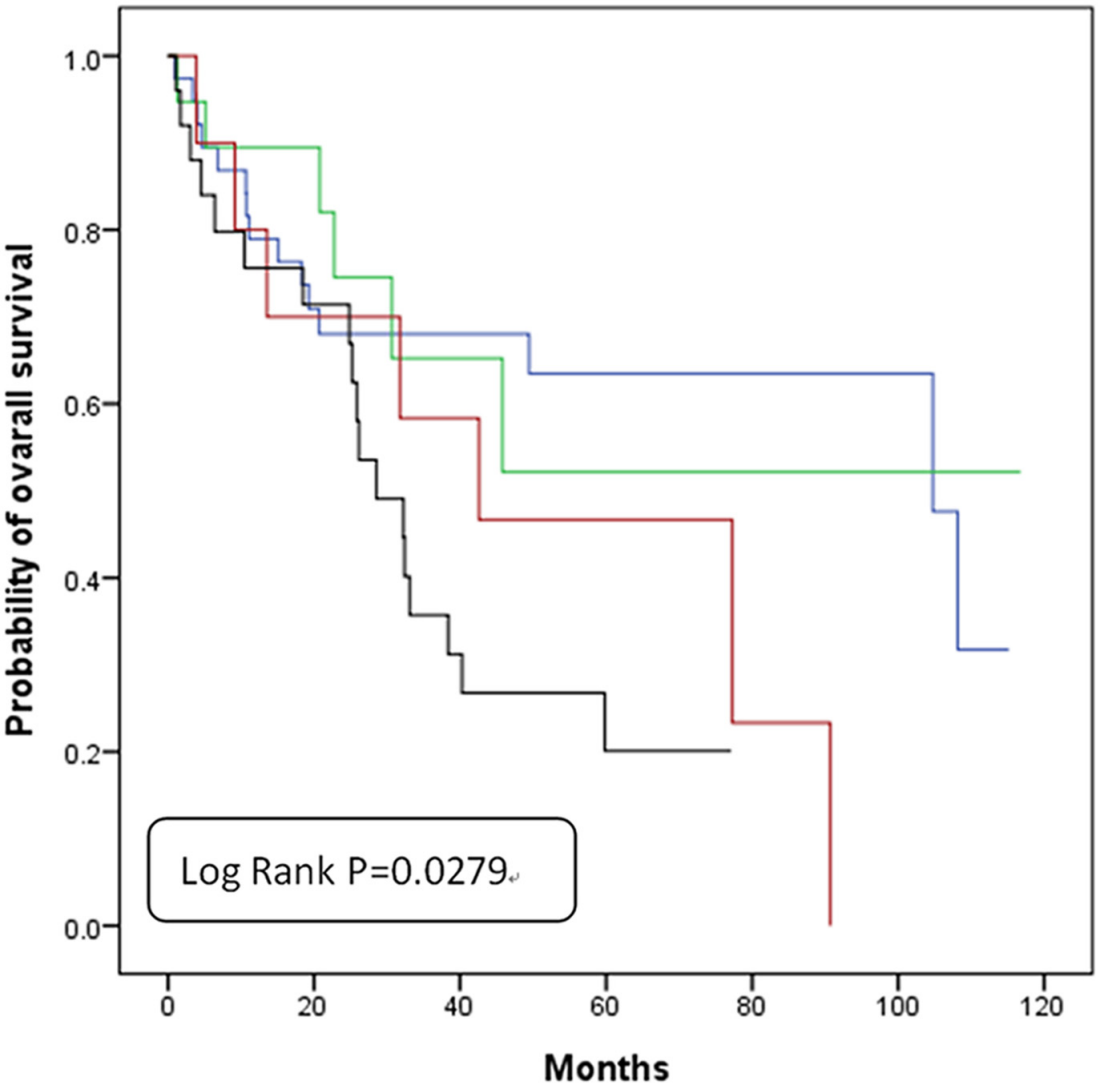
Overall survival of lung adenocarcinoma patients based on expression or lack of expression of FEN1 and RAD54B expressions. Blue line: FEN1(-) and RAD54B(-); Green line: FEN1(+) and RAD54B(-); Red line: FEN1(-) and RAD54B(+); Black line: FEN1(+)and RAD54B(+).

We also examined the prognostic role of each gene individually. As shown in Table 4, RAD54B but not FEN1 was an independent prognostic marker in our study (Multivariate analysis: HR = 2.20, 95% CI = 1.16–4.02, P = 0.0165 for RAD54B; HR = 1.47, 95% CI = 0.80–2.69, P = 0.2148 for FEN1, Table 4). Patients with expression of both FEN1 and RAD54B had significantly poor prognosis (Multivariate analysis: HR = 2.35, 95% CI = 1.13–4.91, P = 0.0230, Table 4). The 5-year survival rate was also significantly lower in patients with expression of both genes when compared with the other groups with only single gene expression (Fig 2). These results suggested that combined overexpression of FEN1 and RAD54B might be an independent marker for poor prognosis in patients with lung adenocarcinoma.

**Table 4:** Univariate and multivariate analysis of FEN1 and RAD54B in lung adenocarcinoma patients.

	Death cases(n = 46)	Survival cases (n = 47)	Univariate HR (95% CI)	P value	Multivariate HR (95% CI)*	P value
FEN1 nucleus, n (%)						
0	22 (47.8)	26 (55.3)	1.00		1.00	
1+2	24 (52.2)	21 (44.7)	1.52 (0.84–2.75)	0.1675	1.47 (0.80–2.69)	0.2148
RAD54B nucleus, n (%)						
0	21 (45.7)	37 (78.7)	1.00		1.00	
1+2	25 (54.4)	10 (21.3)	2.39 (1.31–4.37)	0.0046	2.20 (1.16–4.02)	0.0165
FEN1/ RAD54B, n (%)						
FEN1—and RAD54B -	15 (32.6)	23 (48.9)	1.00		1.00	
FEN1 + and RAD54B -	6 (13.0)	14 (29.8)	0.98 (0.38–2.56)	0.9732	0.87 (0.32–2.37)	0.7910
FEN1—and RAD54B +	7 (15.2)	3 (6.4)	1.98 (0.79–4.92)	0.1431	1.74 (0.68–4.46)	0.2487
FEN1 + and RAD54B +	18 (39.1)	7 (14.9)	2.60 (1.27–5.33)	0.0093	2.35 (1.13–4.91)	0.0230

## Discussion

In this study, we investigated the protein level rather than the combination of mutations of 24 SL-paired genes using 22 different biomarkers in lung adenocarcinoma cancer patients (S1 Table). Of these, only FEN1 and RAD54B were significantly related to the prognosis (Table 4 and Fig 2). Patients with expression of both FEN1 and RAD54B were more prone to have an advanced N stage (Table 3). These results suggest that intensive follow up and targeted therapy might improve clinical outcome for patients who show expression of both FEN1 and RAD54B.

FEN1, a member of the Rad2 nuclease family, has multiple structure-specific nuclease activities for DNA replication and repair [28]. FEN1 plays a key role in Okazaki fragment maturation, base excision repair, and maintenance of telomere stability and apoptosis [29, 30]. FEN1 in complex with WRN employs the GEN activity which is required for efficient telomere replication and suppression of fragile telomeres in mouse model containing E359K mutation, the protein-protein domain of FEN1. [31]. In mouse model, haploinsufficiency of FEN1 leads to rapid tumor progression with increased numbers of adenocarcinoma and decreased survival [32]. Also, FEN-1 mutant mouse showed a high risk of developing lung cancer upon exposure to B[α]P-containing agents such as tobacco smoke [33]. FEN1 plays an essential role in DNA replication; consequently, high levels of FEN1 are believed necessary to support hyperproliferation of cancer cells [29]. Many cancers also show high levels of FEN1 expression, which in some cases is correlated with tumor aggression. In breast and ovarian epithelial cancer, FEN1 protein expression is linked to high grade, high stage and poor survival [34]. For these cases, FEN1-specific inhibitors may have chemotherapeutic potential [35].

RAD54B, a DNA repair and recombination protein, shares similarity with *Saccharomyces cerevisiae* RAD54 [36]. RAD54B serves as a scaffold in the DNA damage response that limits checkpoint strength of both the G1/S and G2/M checkpoints [37]. RAD54B promotes mutagenic adaptation to the G2/M checkpoint and enhances p53 ubiquitination through recruiting MDMX to MDM2 and promoting MDM2–MDMX heterodimerization [37]. RAD54B is highly expressed in the testis and spleen, which suggests active roles in meiotic and mitotic recombination [38, 39]. Homozygous mutations and high expression level of this gene were observed in primary colon cancer, suggesting that some cancers arise through alterations of the RAD54B function [40–42].

Genomic instability affects the expression levels of both oncogene and tumor suppressor genes. Gene interaction creates very complicated networks, so identification of pairs of SL genes in cells is a reasonable approach [11, 43]. SL has been observed in a RAD54B-deficient human colorectal cancer cell line following iatrogenic reduction of FEN1 expression [40]. Therefore, FEN1 and RAD54B could conceivably have overlapping molecular functions involving DNA repair suggestive of SL potential. The observed influence of expression of these two unlinked genes on patient survival in our study could guide the screening of potential anticancer drugs.

Abdel-fatah and colleagues investigated this relationship in a cohort of gastric cancer patients and found a correlation of high expression of FEN1 and lymph node positive disease with poor disease survival [44]. Interestingly, we saw a similar significant correlation between the synergic effect of FEN1 and RAD54B and lymph node metastasis and poor patient survival in our study. However, there are limitations of our study including limited sample size and region source of cases. The follow-up period for our patients was limited, so the long term effect on survival is not known. Smoking history was evaluated in only a few patients so the impact of smoking factors was ignored in our study, which could be a potential limitation. The tissue cores, due to the limitations of tissue microarrays, cannot represent the whole tumor condition, so further and more complete studies are still needed in the future. Otherwise, confirm the protein expression level and specificity of antibody with other clones of antibody should also be considered.

Finding new cancer therapies through a SL mechanism might reveal treatments with greater specificity and fewer complications. Research on SL could result in the development of innovative screening methods and highly effective anticancer therapy drugs. In this study, we did not focus on the role of combination of mutations of SL genes but the overexpression of paired gene in protein level. The study design did not follow the concept of SL but could provide evidence that the concordant overexpression of SL genes in protein level was associated with clinical outcome. The concept of using IHC staining to investigate the prognostic role of synthetic lethal genes was performed previously in colorectal cancer [11]. This approach can be applied to several cancers to predict their unique or common SL pairs and prognosis markers [11]. Also, the contribution of combination of mutations of SL genes in predicting prognosis should be further validated. In present study, the overexpression of FEN1 and RAD54B in poorly prognostic lung adenocarcinoma suggests that these two genes might be potential markers for selecting patients at high risk and could be used in the development of effective personalized therapy.

## Supporting Information

S1 Table Paired synthetic lethal genes measured in this study. (DOCX)
