# Supplementary material for: The Overexpression of FEN1 and RAD54B May Act as Independent Prognostic Factors of Lung Adenocarcinoma
Source: PLoS One. 2015 Oct 2;10(10):e0139435. doi: 10.1371/journal.pone.0139435 (PMC4592204; doi:10.1371/journal.pone.0139435)
Supplement: S1 Table — (DOCX) [file pone.0139435.s001.docx]

Supplementary Table 1. Paired synthetic lethal genes measured in this study.

|  | Gene | Antibody | Gene | Antibody | Reference |
| --- | --- | --- | --- | --- | --- |
| Pair 1 | FEN1 | Abcam, ab462 | RAD54B | Santa cruz, sc-101234 | [[1](#_ENREF_1)] |
| Pair 2 | BRCA2 | Santa cruz, sc-28235 | PARP1 | Santa cruz, sc-8007 | [[2](#_ENREF_2)] |
| Pair 3 | TP53 | Santa cruz, sc-1311 | SGK2 | Abcam, ab71616 | [[3](#_ENREF_3)] |
| Pair 4 | BRCA1 | Abcam, ab48790 | PARP1 | Santa cruz, sc-8007 | [[2](#_ENREF_2)] |
| Pair 5 | MSH2 | Abcam, ab52266 | POLB | Abcam, ab26343 | [[4](#_ENREF_4)] |
| Pair 6 | MYC | Santa cruz, sc-40 | ARK2 | Santa cruz, sc-25426 | [[5](#_ENREF_5)] |
| Pair 7 | BCR | Abcam, ab40779 | WNT5A | Abcam, ab72583 | [[6](#_ENREF_6)] |
| Pair 8 | RB1 | Santa cruz, sc-102 | SKP2 | Abcam, ab68455 | [[7](#_ENREF_7)] |
| Pair 9 | CKIε | Santa cruz, sc-6471 | β-catenin | BD, 610153 | [[8](#_ENREF_8)] |
| Pair 10 | ABL1 | Abcam, ab15130 | WNT5A | Abcam, ab72583 | [[6](#_ENREF_6)] |
| Pair 11 | NOTCH | Abcam, ab27526 | EGFR | Leica, NCL-L-EGFR-384 | [[9](#_ENREF_9)] |
| Pair 12 | E-cadherin | BD, 610181 | Nmi | Santa cruz, sc-22819 | [[10](#_ENREF_10)] |

**References**

1. McManus KJ, Barrett IJ, Nouhi Y, Hieter P. Specific synthetic lethal killing of RAD54B-deficient human colorectal cancer cells by FEN1 silencing. Proc Natl Acad Sci U S A. 2009;106(9):3276-81. Epub 2009/02/17. doi: 10.1073/pnas.0813414106

0813414106 [pii]. PubMed PMID: 19218431; PubMed Central PMCID: PMC2651317.

2. Dedes KJ, Wilkerson PM, Wetterskog D, Weigelt B, Ashworth A, Reis-Filho JS. Synthetic lethality of PARP inhibition in cancers lacking BRCA1 and BRCA2 mutations. Cell Cycle. 2011;10(8):1192-9. Epub 2011/04/14. doi: 15273 [pii]. PubMed PMID: 21487248; PubMed Central PMCID: PMC3117132.

3. Baldwin A, Grueneberg DA, Hellner K, Sawyer J, Grace M, Li W, et al. Kinase requirements in human cells: V. Synthetic lethal interactions between p53 and the protein kinases SGK2 and PAK3. Proc Natl Acad Sci U S A. 2010;107(28):12463-8. Epub 2010/07/10. doi: 10.1073/pnas.1007462107

1007462107 [pii]. PubMed PMID: 20616055; PubMed Central PMCID: PMC2906576.

4. Martin SA, McCabe N, Mullarkey M, Cummins R, Burgess DJ, Nakabeppu Y, et al. DNA polymerases as potential therapeutic targets for cancers deficient in the DNA mismatch repair proteins MSH2 or MLH1. Cancer Cell. 2010;17(3):235-48. Epub 2010/03/17. doi: 10.1016/j.ccr.2009.12.046

S1535-6108(10)00037-1 [pii]. PubMed PMID: 20227038; PubMed Central PMCID: PMC2845806.

5. Yang D, Liu H, Goga A, Kim S, Yuneva M, Bishop JM. Therapeutic potential of a synthetic lethal interaction between the MYC proto-oncogene and inhibition of aurora-B kinase. Proc Natl Acad Sci U S A. 2010;107(31):13836-41. Epub 2010/07/21. doi: 10.1073/pnas.1008366107

1008366107 [pii]. PubMed PMID: 20643922; PubMed Central PMCID: PMC2922232.

6. Tiong KL, Chang KC, Yeh KT, Liu TY, Wu JH, Hsieh PH, et al. CSNK1E/CTNNB1 are synthetic lethal to TP53 in colorectal cancer and are markers for prognosis. Neoplasia. 2014;16(5):441-50. Epub 2014/06/21. doi: 10.1016/j.neo.2014.04.007. PubMed PMID: 24947187; PubMed Central PMCID: PMC4198690.

7. Bauzon F, Zhu L. Racing to block tumorigenesis after pRb loss: an innocuous point mutation wins with synthetic lethality. Cell Cycle. 2010;9(11):2118-23. Epub 2010/05/28. PubMed PMID: 20505340; PubMed Central PMCID: PMC3044209.

8. Kim SY, Dunn IF, Firestein R, Gupta P, Wardwell L, Repich K, et al. CK1epsilon is required for breast cancers dependent on beta-catenin activity. PloS one. 2010;5(2):e8979. Epub 2010/02/04. doi: 10.1371/journal.pone.0008979. PubMed PMID: 20126544; PubMed Central PMCID: PMC2813871.

9. Dong Y, Li A, Wang J, Weber JD, Michel LS. Synthetic lethality through combined Notch-epidermal growth factor receptor pathway inhibition in basal-like breast cancer. Cancer Res. 2010;70(13):5465-74. Epub 2010/06/24. doi: 10.1158/0008-5472.CAN-10-0173

0008-5472.CAN-10-0173 [pii]. PubMed PMID: 20570903.

10. Telford BJ, Chen A, Beetham H, Frick J, Brew TP, Gould CM, et al. Synthetic Lethal Screens Identify Vulnerabilities in GPCR Signaling and Cytoskeletal Organization in E-Cadherin-Deficient Cells. Molecular cancer therapeutics. 2015;14(5):1213-23. Epub 2015/03/18. doi: 10.1158/1535-7163.MCT-14-1092. PubMed PMID: 25777964.
